# Human Amniotic Mesenchymal Stromal Cells Promote Bone Regeneration via Regulating Ameloblastoma-Derived-Bone Marrow Mesenchymal Cells Crosstalk and Autophagy in Ameloblastoma Microenvironment

**DOI:** 10.1007/s13770-025-00789-8

**Published:** 2026-01-24

**Authors:** Yuhuan Xiao, Xiaofeng Fu, Weina Zhou, Jin Li, Bin Yan, Fei Jiang

**Affiliations:** 1 Department of Orthodontics, The Affiliated Stomatological Hospital of Nanjing Medical University, Nanjing, 210029 Jiangsu People’s Republic of China; 2 State Key Laboratory Cultivation Base of Research, Prevention and Treatment for Oral Diseases (Nanjing Medical University), Nanjing Medical University, No. 140 Hanzhong Road, Nanjing, 210029 Jiangsu People’s Republic of China; 3 Jiangsu Province Engineering Research Center of Stomatological Translational Medicine (Nanjing Medical University), Nanjing, 210029 Jiangsu People’s Republic of China; 4 Department of General Dentistry, State Key Laboratory Cultivation Base of Research, Prevention and Treatment for Oral Diseases, Affiliated Hospital of Stomatology, Nanjing Medical University, No. 140 Hanzhong Road, Nanjing, 210029 Jiangsu People’s Republic of China; 5 Department of Stomatology, The Affiliated Children’s Hospital of Jiangnan University, Wuxi, 214000 Jiangsu People’s Republic of China; 6 Department of Temporomandibular Joint, The Affiliated Stomatological Hospital of Nanjing Medical University, Nanjing, 210029 Jiangsu People’s Republic of China

**Keywords:** M-AMCs, HAMSCs, Autophagy, Ameloblastoma, Bone formation

## Abstract

**Background:**

Growing evidence validates the vital function of mesenchymal stem cells (MSCs) in tumor development. Our previous findings have illustrated the role of MSCs in the invasion and recurrence of ameloblastoma. Stem cells can be transplanted to release paracrine factors in the tumor microenvironment (TME) to inhibit tumor progression and recurrence. The paracrine function of human amniotic mesenchymal stromal cells (HAMSCs) benefits bone regeneration. However, the dual function of HAMSCs in inhibiting tumor progression and promoting bone regeneration in the TME remains unknown.

**Methods:**

To analyze the role of HAMSCS in the cross-talk between mesenchymal ameloblastoma-derived cells (M-AMCs), human bone marrow mesenchymal stem cells (HBMSCs), and HAMSCs, an *in vitro* co-culture system of M-AMCs, HBMSCS, and HAMSCS was prepared. An *in vivo* ectopic transplantation model was employed further to detect the therapeutic effect of HAMSCs on bone regeneration.

**Results:**

A high-level basal autophagy was detected in the stroma of ameloblastoma. In the *in vitro* co-culture models, M-AMCs suppressed the proliferation, differentiation, migration, and autophagy of HBMSCs, and conversely, HBMSCs promoted the above phenotypes of M-AMCs. HAMSCs promoted the proliferation, differentiation, migration and autophagy of the co-cultured HBMSCs. Additionally, HAMSCs mediated the cross-talk between M-AMCs and HBMSCs. The *in vivo* ectopic transplantation model indicated that transplanted HAMSCs promoted bone regeneration by inhibiting the growth of M-AMCs and enhancing autophagy, as well as osteogenesis in bone defects of mice.

**Conclusions:**

The interaction of M-AMCs and HBMSCs may be associated with ameloblastoma recurrence. HAMSCs regulate the cross-talk between M-AMCs and HBMSCs to increase the autophagic level in the TME, thus inhibiting the progression and recurrence of ameloblastoma and promoting bone regeneration. Therefore, HAMSC-based therapy provides an alternative to facilitate bone regeneration and repair of ameloblastoma-induced bone defects.

## Introduction

Ameloblastoma constitutes approximately 14% of jaw tumors and poses a substantial clinical challenge. Despite its histologically benign nature, it demonstrates locally aggressive behavior by invading the alveolar bone and causing significant destruction [1–3]. Recurrence rates following conservative treatment range from 55 to 90%. Even after radical resection, 60% of patients develop permanent jaw dysfunction, typically presenting as impaired mastication or facial deformities resulting from bone defects. Existing therapies fail to address two critical clinical needs: effective control of tumor recurrence and jaw function preservation. Most mechanistic studies have concentrated on individual drivers, such as SMO mutations and CD44 + cancer stem cells, while neglecting the pivotal role of tumor stroma-bone crosstalk in facilitating tumor invasiveness and bone destruction [4]. This mechanism remains unreported, and dual-effect therapeutic strategies have not been thoroughly investigated [5].

Autophagy has emerged as a central regulator of this crosstalk, although its function in ameloblastoma remains paradoxical. In bone marrow mesenchymal stem cells (BMSCs), autophagy supplies energy for osteogenic differentiation, as demonstrated by the observation that knockdown of autophagy-related gene 5 (ATG5) or Beclin1 directly impairs osteogenic potential, which is essential for the repair of tumor-induced bone defects [6–8]. Conversely, recent evidence indicates that autophagy in M-AMCs enhances their capacity to degrade bone matrix. This phenomenon constitutes an “autophagy dichotomy”: autophagy promotes repair in BMSCs but facilitates invasion in tumor cells within the TME. Selective modulation of autophagy in these distinct cell types is a prerequisite for tumor control and bone regeneration.

Stem cell-based therapies hold promise as a solution, and mesenchymal stem cells (MSCs) exhibit tumor-homing and paracrine properties that suppress malignancy [9, 10]. Human amniotic mesenchymal stem cells (HAMSCs) stand out for strong clinical translatability: they are easily extracted, have an abundant supply and superior paracrine capacity, avoid immune rejection, and raise no ethical concerns [11]. Unlike non-specific autophagy inhibitors such as chloroquine, which disrupt normal bone homeostasis, HAMSCs have been shown to suppress autophagy in hepatoma cells while activating it in osteoblasts [12–15]. This hints at their potential to resolve the “autophagy dichotomy” in ameloblastoma. However, whether HAMSCs can regulate the crosstalk between M-AMCs and HBMSCs via autophagy remains unexplored. Additionally, whether this regulatory effect can achieve tumor inhibition and bone repair remains unstudied.

**Fig. 1:**
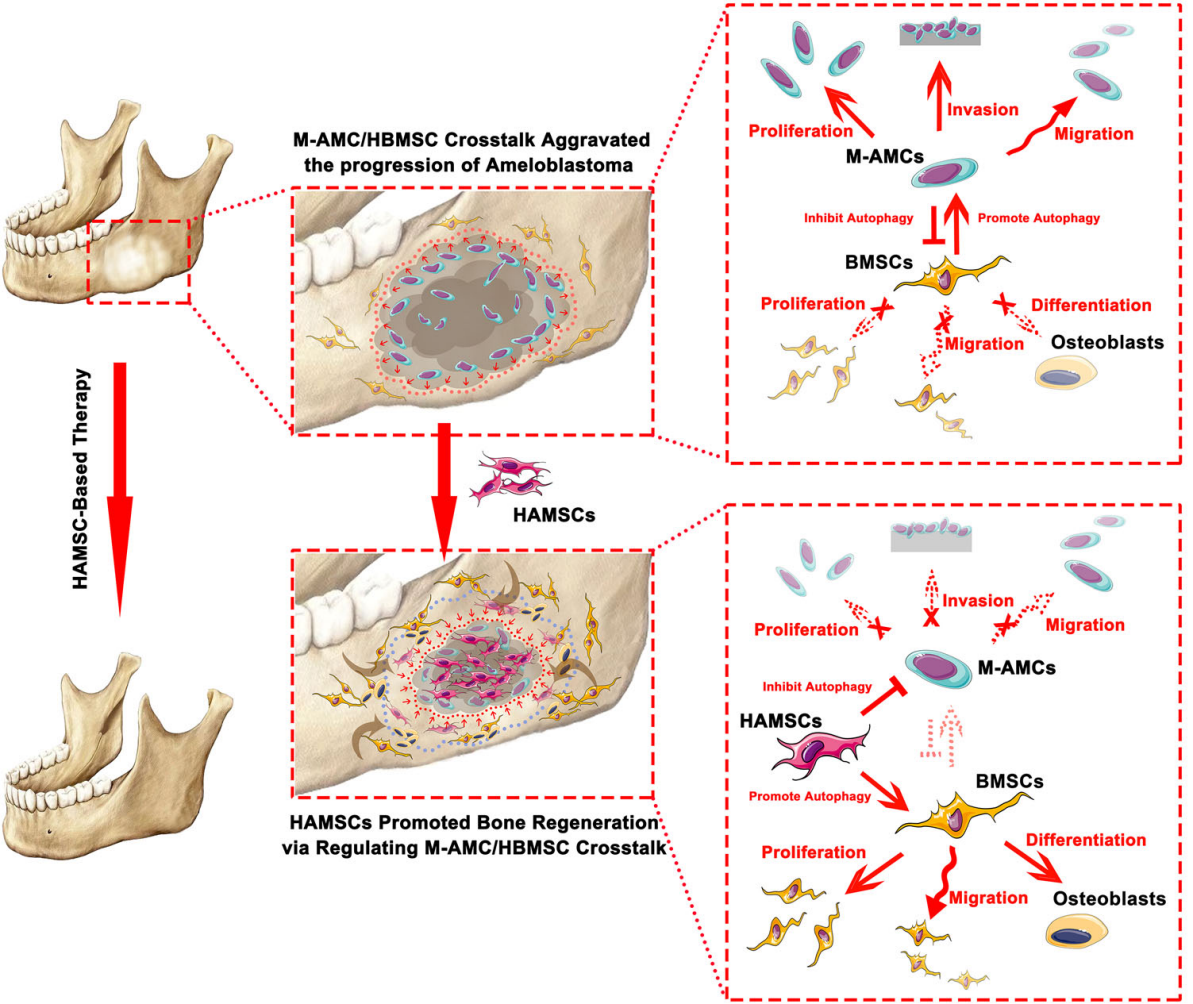
The schematic diagram of HAMSC-based therapy for promoting the bone regeneration in ameloblastoma -induced bone defects via regulating the cross-talk between M-AMCs and HBMSCs

To elucidate the role of HAMSCs in regulating tumor-stromal crosstalk within the ameloblastoma TME, a triple co-culture system comprising HAMSCs, M-AMCs, and HBMSCs was established (Fig. 1). A subcutaneous model was also employed for single-variable analysis, thereby minimizing confounding factors inherent in orthotopic models, such as the complex bone microenvironment. Centred on autophagy, this study aims to verify three hypotheses: HAMSCs suppress the invasive capacity of M-AMCs by inhibiting their pro-tumor autophagy, enhance the osteogenic potential of HBMSCs by activating their pro-repair autophagy, and mediate these dual effects through paracrine signals such as TGF-β1. This research provides experimental evidence to address the clinical need for controlling ameloblastoma recurrence while promoting jaw bone regeneration.

## Materials and methods

### Specimen collection

Ameloblastoma specimens were collected from patients with ameloblastoma who were admitted and pathologically confirmed at the Affiliated Stomatological Hospital of Nanjing Medical University from January 2020 to December 2022. Healthy alveolar bone tissues were collected during the same period as negative controls. This study was approved by the ethics committee (No. PJ2021-153-001), and written informed consent was obtained.

### Extraction of M-AMCs and cell identification

M-AMCs were extracted from ameloblastoma tissue blocks by trypsin digestion. Fresh medium was replaced at intervals of three days. Cell colonies (> 50 cells) fixed in 4% paraformaldehyde and stained with 0.15% crystal violet were observed under a light microscope. M-AMCs were routinely passaged, and the third-generation (P3) M-AMCs were prepared for the single-cell suspension. After washing in PBS, the single-cell suspension was incubated with anti-CD44 (PE), anti-CD73 (APC), anti-CD90 (FITC), anti-CD105 (CY5), and anti-CD133 (APC) (BioLegend, USA). Flow cytometry was performed on the BD FACS Calibur (Becton, Dickinson and Company, USA) for cell identification.

### Cell culture

M-AMCs extracted from ameloblastoma tissues, and HAMSCs and HBMSCs (Chinese Academy of Sciences) were cultured in α-MEM containing 10% fetal bovine serum (FBS) and 1% penicillin–streptomycin in a humidified incubator with 5% CO_2_ at 37 °C.

### Co-culture systems

Transwell cocultures were performed as previously described. In the co-culture system of HBMSCs or HAMSCs and M-AMCs, HBMSCs/HAMSCs were implanted on the 24-well plate, and M-AMCs were seeded in the Transwell insert. The co-culture system involving HBMSCs, HAMSCs, and M-AMCs was similarly prepared. Fresh medium was replaced every three days, and cell supernatant was collected for further use.

### Immunohistochemistry

Paraffin-embedded tissue blocks were subjected to fixation, dehydration, and antigen retrieval, and then incubated with the anti-ATG5 antibody (1:100; Abcam, UK) and anti-Vimentin antibody (1:200; Cell Signaling Technology, USA). After counterstaining, dehydrating, and stabilizing with mounting medium, the stained pictures were captured under the microscope.

### EdU assay

Cell proliferation was examined using the BeyoClick EdU kit (Beyotime Biotechnology, China). Briefly, cells seeded in the 24-well plate were incubated with 200 μL of fresh medium containing 100 μM 5-ethynyl-2′-deoxyuridine (EdU) for 2 h at 37 °C, fixed with 4% paraformaldehyde, and stained with Hoechst 33,342 and the Apollo reaction cocktail. Three random images per sample were captured to calculate the percentage of EdU-positive cells.

### Osteogenic differentiation

HBMSCs seeded in a 24-well plate were cultured in the osteogenic induction medium, and Transwell inserts implanted with M-AMCs and HAMSCs were placed in the plate. On day 7, cells were fixed with 4% paraformaldehyde, incubated in an alkaline solution at room temperature (RT) for 20 min, and stained with NBT/BCIP (Beyotime Biotechnology). On day 14, cells were stained with 1% (w/v) Alizarin Red S (ARS) (Beyotime Biotechnology) for 10 min at RT. Images were captured under a light microscope.

### Transwell assay

Transwell assay using the 8.0 μm pore polycarbonate membrane inserts was performed to examine cell migration and invasion. Briefly, M-AMCs, HBMSCs, or HAMSCs were seeded in the lower chamber of the 24-well plate, and HBMSCs and M-AMCs at 2 × 10^4^ cells/well were seeded into the upper chambers in serum-free medium. After 24 h and 72 h, unpenetrated cells were wiped off. Migratory cells that had penetrated the lower chamber were fixed in 4% paraformaldehyde, stained with 0.15% crystal violet dye solution, and captured for counting cell number. Invasion assay was performed using the 8.0 μm pore polycarbonate membrane inserts precoated with 60 μL of Matrigel.

### Immunofluorescence

Cells or tissue blocks were fixed in 4% paraformaldehyde, permeabilized with 0.5% Triton X-100, and blocked with 5% sheep serum for 60 min. Immunofluorescence labelling of anti-ALP antibody (10 μg/ml; RD systems, UK) and anti-ATG5 antibody (1:150; Abcam) was performed overnight at 4 °C. The other day, they were incubated with Alexa Fluor® 488 and 594 secondary antibodies (1:200; Invitrogen, USA) in the dark for 2 h. Cell nuclei were stained for 5 min at RT with DAPI (Sigma, USA). Immunofluorescence staining was visualized under a fluorescent microscope (Olympus Corporation, Japan).

### Western blot

Total proteins were extracted from cells, quantified using the BCA Protein Assay Kit (Beyotime Biotechnology), prepared into samples, separated on the SDS-PAGE and transferred to PVDF membranes. After immersing in 10% non-skim milk for 2 h, the membranes were incubated with anti-ATG5 (1:1000; Abmart, China), anti-Beclin1 (1:1000; Abmart), anti-LC3 II/I (1:2000; Abcam), anti-ALP (1:1000; Affinity, China), anti-RUNX2 (1:1000; Cell Signaling Technology), anti-OSX (1:1000; Abmart), anti-OPN (1:1000; Boster, China), anti-MMP2 (1:1000; Abcam) and anti-MMP9 (1:1000; Abcam) overnight at 4 °C. Immunoblots were incubated with secondary antibodies for 2 h at RT, and visualized using the enhanced chemiluminescent method.

### *In vivo* ectopic transplantation model

Silk scaffolds were prepared using the 5–6% (w/v) silk solution, followed by lyophilization, autoclavation at 121 °C for 20 min, and trimming (5 mm in diameter and 3 mm in height). BALB/c nude mice were intravenously anesthetized with pentobarbital sodium (1.5 mg/kg). Silk scaffolds, silk-BMP-2 scaffolds (0.3 mg/mL BMP-2 as the positive control), silk scaffolds seeded with 20 µL of 0.5 × 10^6^ M-AMCs, and scaffolds seeded with M-AMCs co-cultured with HAMSCs were then subcutaneously transplanted into the back of nude mice (n = 6 per group). They were sacrificed four weeks later, and silk scaffolds were collected, fixed in 4% paraformaldehyde, and prepared into paraffin-embedded tissue blocks (4 mm in thickness). Animal procedures were approved by the Institutional Animal Care and Use Committee (IACUC-2005034).

### Statistical analysis

Data were expressed as mean ± standard deviation (SD), and differences among three or more groups were compared by the one-way analysis of variance (ANOVA). A significant difference was determined at *p* < 0.05.

## Results

### Basal autophagy is enhanced in the stroma of ameloblastoma

**Fig. 2:**
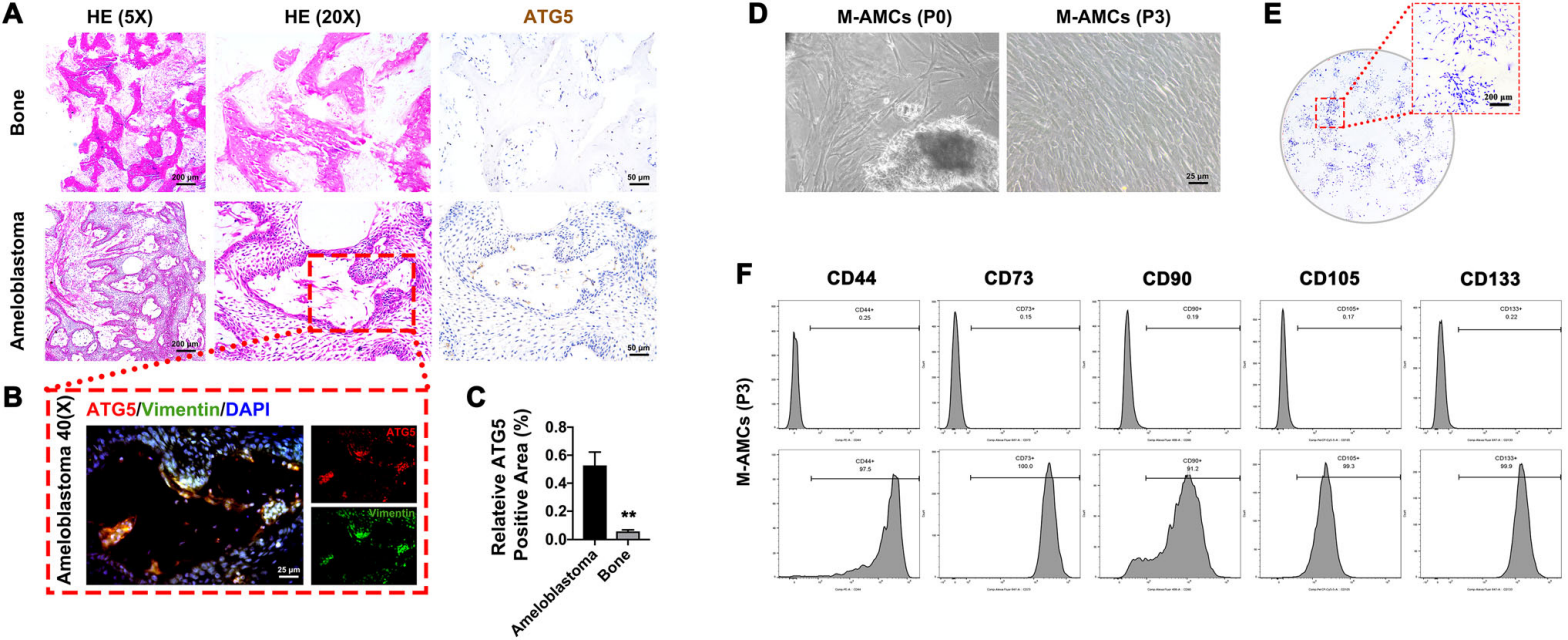
Basal autophagy in ameloblastoma and identification of mesenchymal components in M-AMCs. **A** H&E staining of ATG5 in ameloblastoma and alveolar bone specimens (magnification = 5 × and 20 ×). **B**, **C** Immunofluorescent staining of Vimentin (green) and ATG5 (red) in ameloblastoma specimens (magnification = 40 ×). Cell nuclei were stained with DAPI (blue). **D** Growth state of P0 and P3 M-AMCs (scale bar: 25 μm). **E** Colony formation of M-AMCs (scale bar: 200 μm). **F** Flow cytometry of CD44, CD133, CD73, CD90 and CD105 in M-AMCs. via flow cytometry. **p < 0.01

Growing evidence has supported the role of autophagy in accelerating tumor growth. Here, we detected the positive expression of ATG5 in ameloblastoma specimens and alveolar bone tissues (Fig. 2A–C). The expression level of ATG5 was significantly enhanced in ameloblastoma specimens. Interestingly, ATG5 was co-localized with the mesenchymal marker Vimentin in ameloblastoma specimens, validating the presence of mesenchymal stroma in the components of ameloblastoma (Fig. 2B).

In primary ameloblastoma cells (P0), epithelial components appeared like paving stones while mesenchymal components were spindle-shaped. They proceeded to proliferate and merge progressively with the prolongation of cell culture. After cell passage into the third generation (P3), mesenchymal components were predominant in ameloblastoma cells (Fig. 2D) Colony formation assay consistently proved the similar characteristics between M-AMCs and tumor stem cells (Fig. 2E). Moreover, M-AMCs were positive for mesenchymal markers on the cell surface, including CD44, CD133, CD73, CD90, and CD105 (Fig. 2F). These findings implied that M-AMCs possessed strong colony formation, proliferation, and self-renewal ablilities.

### M-AMCs inhibit the proliferation, differentiation, migration, and autophagy of co-cultured HBMSCs

**Fig. 3:**
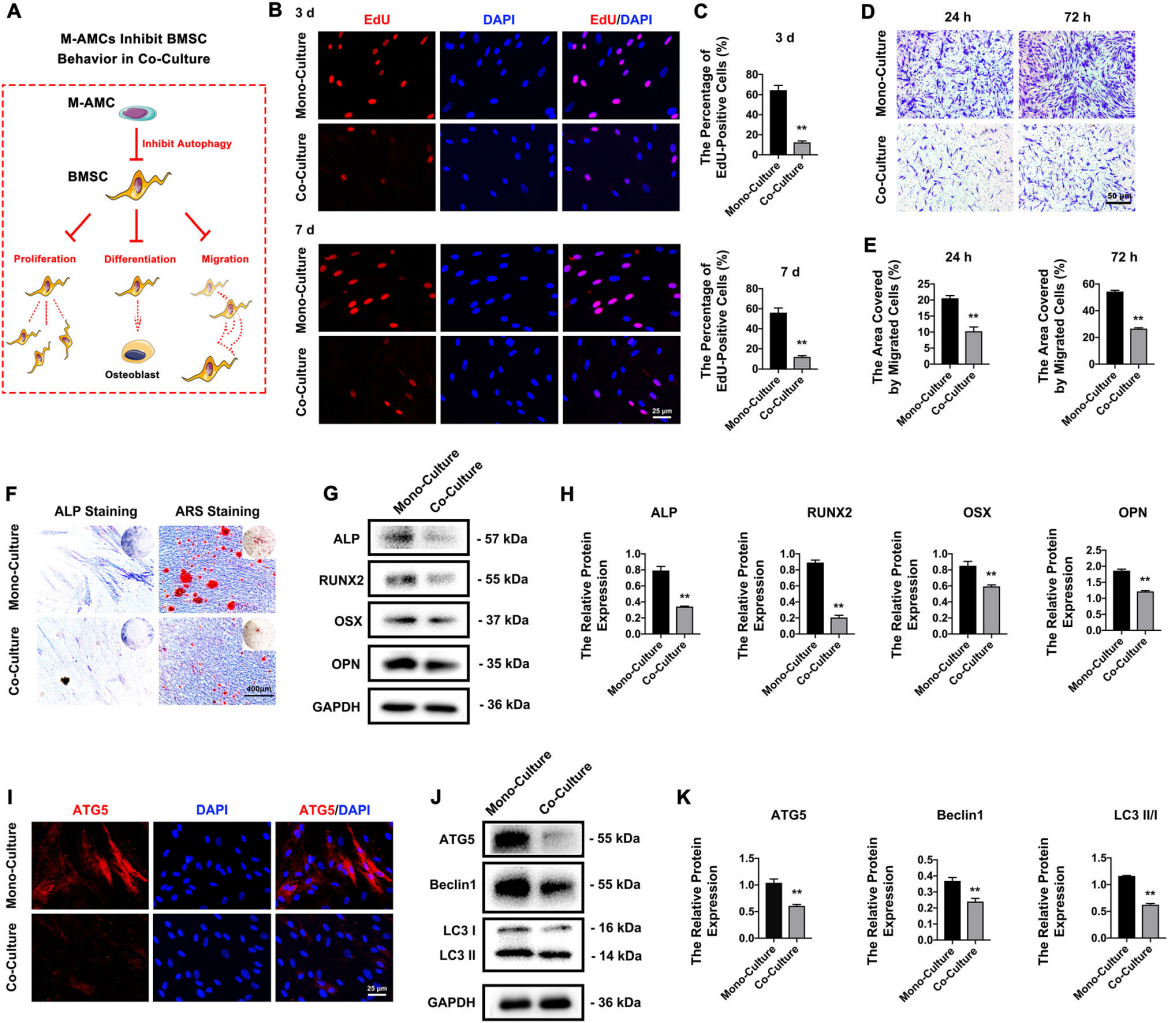
M-AMCs inhibit cell behaviors of co-cultured HBMSCs. **A** A schematic diagram of M-AMCs in inhibiting proliferation, differentiation and migration of co-cultured HBMSCs. **B**, **C** EdU-positive HBMSCs (red) in the mono-culture and co-culture systems (scale bar: 25 μm). Cell nuclei were stained with DAPI (blue). **D**, **E** Transwell migration assay of HBMSCs in the mono-culture and co-culture systems (scale bar: 50 μm). **F** ALP and ARS staining of HBMSCs in the mono-culture and co-culture systems (scale bar: 100 μm). **G**, **H** Protein expressions of ALP, RUNX2, OSX and OPN in HBMSCs of the mono-culture and co-culture systems. **I** Immunofluorescent staining of ATG5 (red) in HBMSCs of the mono-culture and co-culture systems (scale bar: 25 μm). Cell nuclei were stained with DAPI (blue). **J**, **K** Protein expressions of ATG5, LC3 II/I and Beclin1 in HBMSCs of the mono-culture and co-culture systems. **p < 0.01

A co-culture system of M-AMCs and HBMSCs was prepared to investigate the influence of M-AMCs on the behaviors of HBMSCs (Fig. 3A). After 3 days and 7 days of co-culture, the percentage of EdU-positive HBMSCs was significantly lower than that in mono-cultured HBMSCs (Fig. 3B–C). The migratory ability at 24 and 72 h was significantly reduced in co-cultured HBMSCs compared with that in mono-cultured HBMSCs (Fig. 3D). Mineralized nodules formed by HBMSCs in the co-culture system were fewer than those in the mono-culture system, indicating the inhibited osteogenic differentiation (Fig. 3F). Significant downregulations of ALP, RUNX2, OSX, and OPN proved the suppression of osteogenesis in co-cultured HBSMCs (Fig. 3G–H). Immunofluorescence of co-cultured HBMSCs three days after osteogenic induction revealed a significant decrease in the positive expression of ATG5 (Fig. 3I). Moreover, seven days of co-culture significantly downregulated ATG5, Beclin1, and LC3 II/I protein levels in HBMSCs by M-AMCs (Fig. 3J–K). Overall, M-AMCs significantly inhibited the proliferation, differentiation, migration, and autophagy in co-cultured HBMSCs.

### HBMSCs promote the proliferation, migration, invasion, and autophagy of co-cultured M-AMCs

**Fig. 4:**
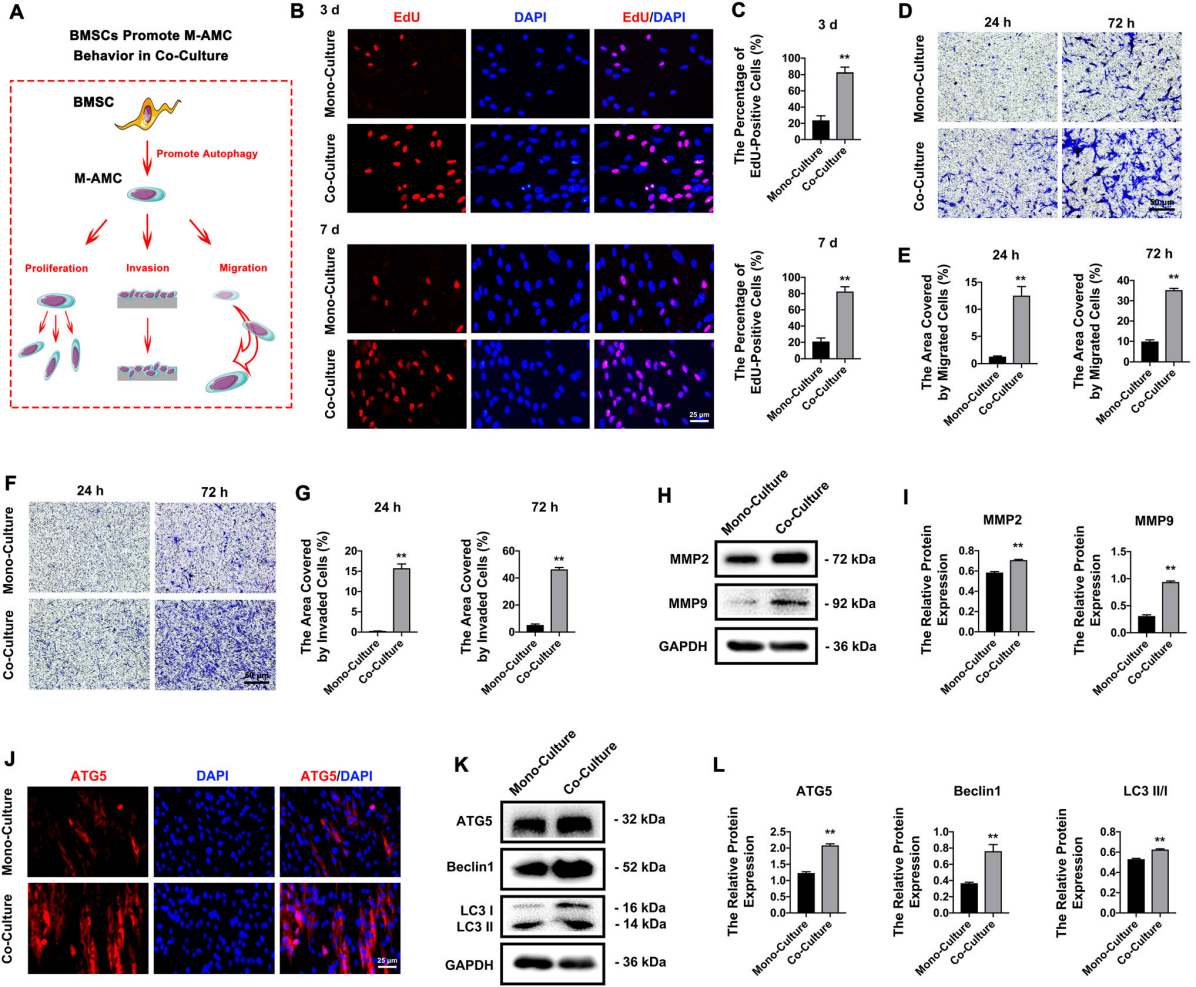
HBMSCs promote cell behaviors of co-cultured M-AMCs. **A** A schematic diagram of HBMSCs in promoting proliferation, invasion and migration of co-cultured M-AMCs. **B**, **C** EdU-positive M-AMCs (red) in the mono-culture and co-culture systems (scale bar: 25 μm). Cell nuclei were stained with DAPI (blue). **D**, **E** Transwell migration assay of M-AMCs in the mono-culture and co-culture systems (scale bar: 50 μm). **F**, **G** Transwell invasion assay of M-AMCs invasion assay in the mono-culture and co-culture systems (scale bar: 50 μm). **H**, **I** Protein expressions of MMP2 and MMP9 in M-AMCs of the mono-culture and co-culture systems. **J** Immunofluorescent staining of ATG5 (red) in M-AMCs of the mono-culture and co-culture systems (scale bar: 25 μm). Cell nuclei were stained with DAPI (blue). **K**, **L** Protein expressions of ATG5, LC3 II/I and Beclin1 in M-AMCs of the mono-culture and co-culture systems. **p < 0.01

To explore the cross-talk between M-AMCs and HBMSCs, we later explored the influence of HBMSCs on the behaviors of M-AMCs in the co-culture system (Fig. 4A). Attributed to the mesenchymal components in M-AMCs, HBMSCs significantly increased the percentages of EdU-positive M-AMCs at 3 days and 7 days of co-culture (Fig. 4B, C), as well as higher migratory (Fig. 4D, E) and invasive capacities (Fig. 4F, G) at 24 h and 72 h. Significantly upregulated MMP2 and MMP9 in co-cultured M-AMCs consistently validated the mesenchymal property in M-AMCs (Fig. 4H, I). In addition, the upregulated ATG5, Beclin1 and LC3 II/I in co-cultured M-AMCs by HBMSCs indicated the enhanced autophagy (Fig. 4K, L). We confirmed that M-AMCs and HBMSCs interacted to regulate their behaviors.

### HAMSCs inhibit the proliferation, migration, invasion, and autophagy of co-cultured M-AMCs

**Fig. 5:**
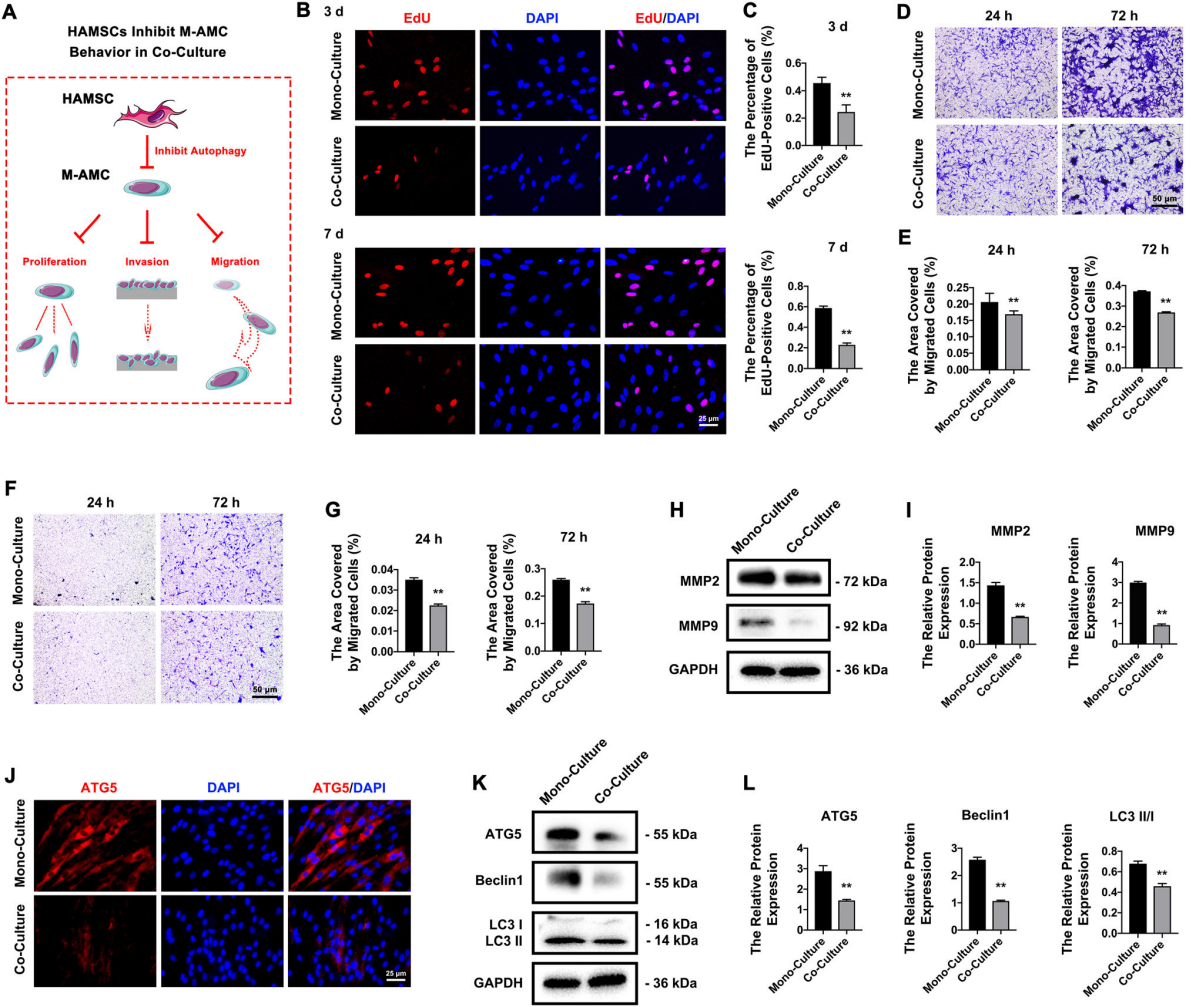
HAMSCs inhibit cell behaviors of co-cultured M-AMCs. **A** A schematic diagram of HAMSCs in promoting proliferation, invasion and migration of co-cultured M-AMCs. (B, C) EdU-positive M-AMCs (red) in the mono-culture and co-culture systems (scale bar: 25 μm). Cell nuclei were stained with DAPI (blue). **D**, **E** Transwell migration assay of M-AMCs in the mono-culture and co-culture systems (scale bar: 50 μm). **F**, **G** Transwell invasion assay of M-AMCs invasion assay in the mono-culture and co-culture systems (scale bar: 50 μm). **H**, **I** Protein expressions of MMP2 and MMP9 in M-AMCs of the mono-culture and co-culture systems. **J** Immunofluorescent staining of ATG5 (red) in M-AMCs of the mono-culture and co-culture systems (scale bar: 25 μm). Cell nuclei were stained with DAPI (blue). **K**, **L** Protein expressions of ATG5, LC3 II/I and Beclin1 in M-AMCs of the mono-culture and co-culture systems. **p < 0.01

Similarly, in the co-culture system of HAMSCs and M-AMCs, the regulatory effects of HAMSCs on the behaviors of co-cultured M-AMCs were examined (Fig. 5A). After 3 days and 7 days of co-culture, the percentage of EdU-positive M-AMCs was significantly reduced by HAMSCs (Fig. 5B, C). Migratory (Fig. 5D, E) and invasive capacities (Fig. 5F, G) were significantly suppressed at 24 h and 72 h in co-cultured M-AMCs. As expected, MMP2 and MMP9 were significantly downregulated in the co-culture system of M-AMCs (Fig. 5H, I). Suppressed autophagy in co-cultured M-AMCs was validated by the less positive staining of ATG5 (Fig. 5J) and lower protein expressions of ATG5, Beclin1, and LC3 II/I in the co-culture system of M-AMCs (Fig. 5K, L). Besides suppressing autophagy, HAMSCs exerted inhibitory effects on the proliferation, migration, and invasion of M-AMCs.

### HAMSCs promote the proliferation, migration, differentiation, and autophagy of co-cultured HBMSCs

**Fig. 6:**
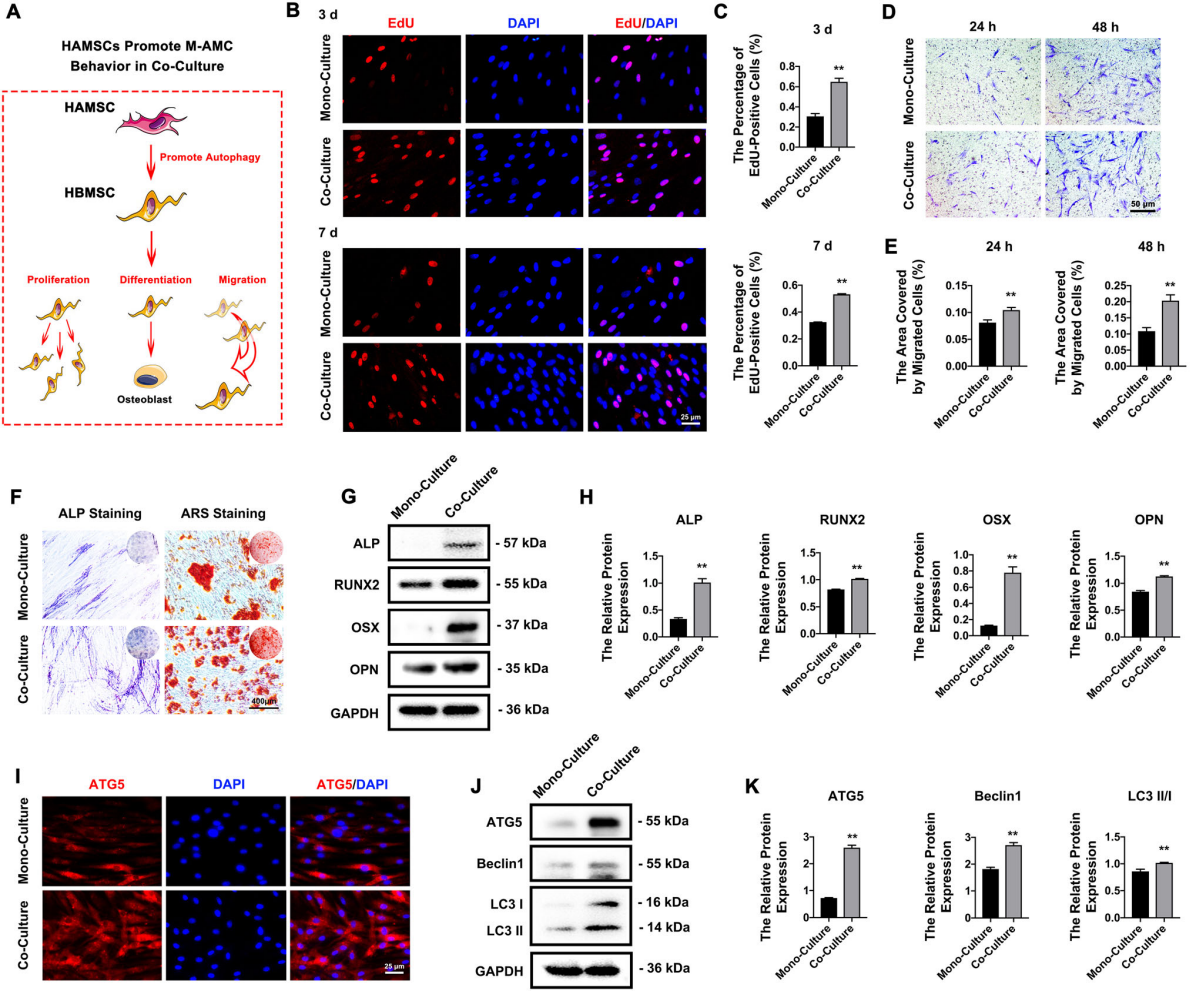
HAMSCs promote cell behaviors of co-cultured HBMSCs. **A** A schematic diagram of HAMSCs in promoting proliferation, differentiation and migration of co-cultured HBMSCs. **B**, **C**) EdU-positive HBMSCs (red) in the mono-culture and co-culture systems (scale bar: 25 μm). Cell nuclei were stained with DAPI (blue). **D**, **E** Transwell migration assay of HBMSCs in the mono-culture and co-culture systems (scale bar: 50 μm). **F** ALP and ARS staining of HBMSCs in the mono-culture and co-culture systems (scale bar: 100 μm). **G**, **H** Protein expressions of ALP, RUNX2, OSX and OPN in HBMSCs of the mono-culture and co-culture systems. **I** Immunofluorescent staining of ATG5 (red) in HBMSCs of the mono-culture and co-culture systems (scale bar: 25 μm). Cell nuclei were stained with DAPI (blue). **J**, **K** Protein expressions of ATG5, Beclin1 and LC3 II/I in HBMSCs of the mono-culture and co-culture systems. **P < 0.01

Notably, HAMSCs are excellent donor cells to promote endogenous bone regeneration by activating the autophagy signalling pathway. When co-cultured with HBMSCs (Fig. 6A), HAMSCs contributed to significantly enhancing the percentage of EdU-positive (Fig. 6B, C) and migratory HBMSCs (Fig. 6D, E). We also observed more pronounced ALP and ARS staining (Fig. 6F) and higher protein expressions of ALP, RUNX2, OSX and OPN (Fig. 6G, H) in co-cultured HBMSCs than mono-cultured HBMSCs, suggesting the accelerated osteogenic differentiation by HAMSCs. In addition, a stronger positive staining of ATG5 (Fig. 6I) and higher protein levels of ATG5, LC3 II/I and Beclin1 (Fig. 6J, K) were indicative of enhanced autophagy in co-cultured HBMSCs.

### Role of HAMSCs in the TME where M-AMCs and HBMSCs co-exist

We inoculated and directly co-cultured HAMSCs, HBMSCs, and M-AMCs to clarify whether HAMSCs can inhibit M-AMCs or enhance HNMSCs in the TME through their excellent paracrine effects.

### HAMSCs inhibit the behaviors of M-AMCs promoted by HBMSCs

**Fig. 7:**
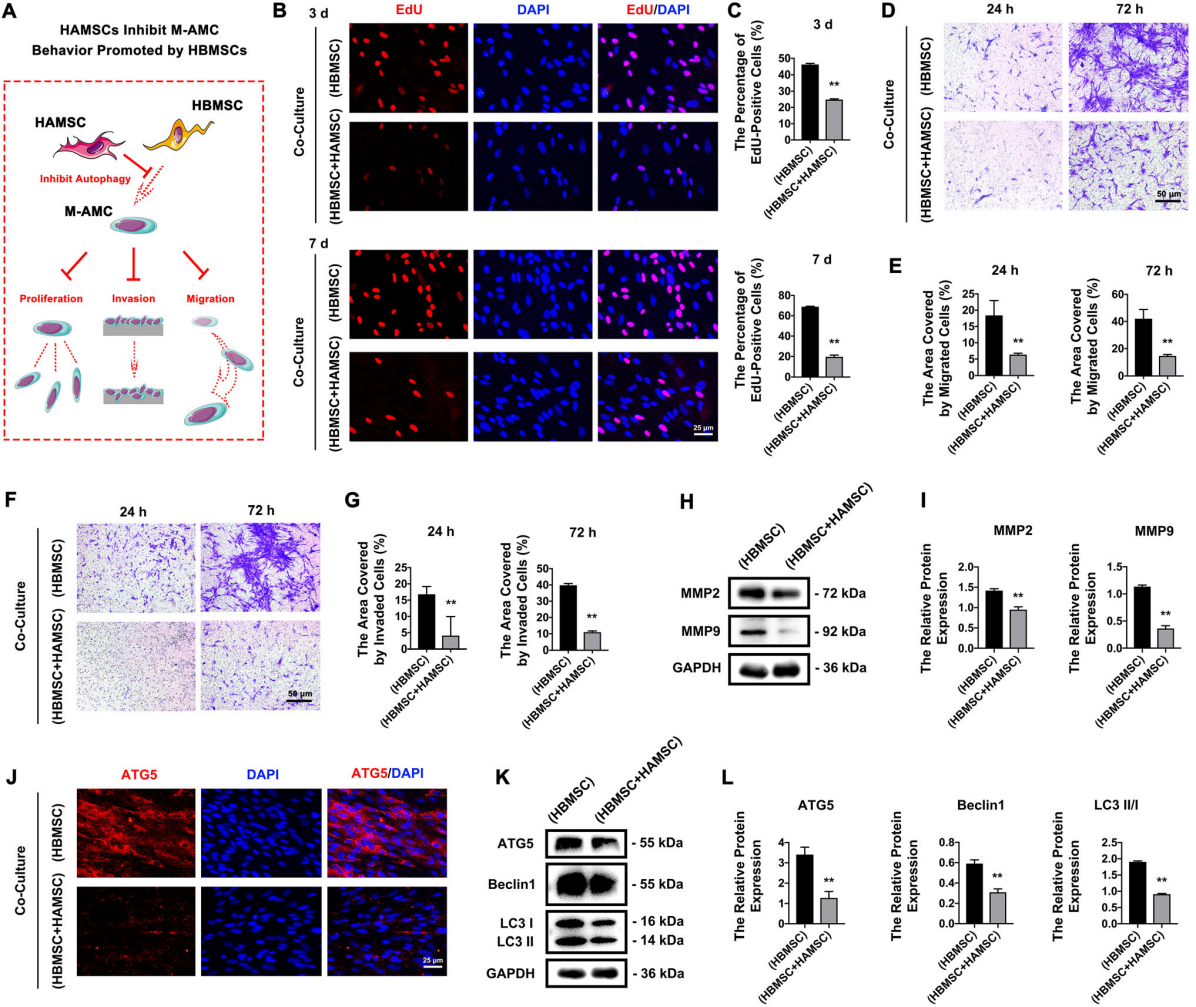
HAMSCs inhibit cell behaviors of M-AMCs promoted by HBMSCs. **A** A schematic diagram of HAMSCs in inhibiting the proliferation, invasion and migration of M-AMCs promoted by HBMSCs. **B**, **C** EdU-positive M-AMCs (red) in HBMSCs/M-AMCs co-culture system and HAMSCs/HBMSCs/M-AMCs co-culture system (scale bar: 25 μm). Cell nuclei were stained with DAPI (blue). **D**, **E** Transwell migration assay of M-AMCs in HBMSCs/M-AMCs co-culture system and HAMSCs/HBMSCs/M-AMCs co-culture system (scale bar: 50 μm). **F**, **G** Transwell invasion assay of M-AMCs in HBMSCs/M-AMCs co-culture system and HAMSCs/HBMSCs/M-AMCs co-culture system (scale bar: 50 μm). **H**, **I** Protein expressions of MMP2 and MMP9 in M-AMCs of HBMSCs/M-AMCs co-culture system and HAMSCs/HBMSCs/M-AMCs co-culture system. **J** Immunofluorescent staining of ATG5 (red) in M-AMCs of HBMSCs/M-AMCs co-culture system and HAMSCs/HBMSCs/M-AMCs co-culture system (scale bar: 25 μm). Cell nuclei were stained with DAPI (blue). **K**, **L** Protein expressions of ATG5, LC3 II/I and Beclin1 in M-AMCs of HBMSCs/M-AMCs co-culture system and HAMSCs/HBMSCs/M-AMCs co-culture system. **p < 0.01

A complicated co-culture system of HAMSCs, M-AMCs, and HBMSCs was prepared to illustrate the role of HAMSCs in regulating the cross-talk between the latter two in the TME of ameloblastoma via the paracrine effect (Fig. 7A). Interestingly, we observed that HAMSCs significantly inhibited the behaviors of M-AMCs promoted by HBMSCs. Compared with those in the co-culture system of HBMSCs and M-AMCs, we detected significantly lower percentage of EdU-positive M-AMCs (Fig. 7B, C) and lower migratory (Fig. 7D, E) and invasive capacities (Fig. 7F, G) in co-cultured M-AMCs with HAMSCs and HBMSCs. Significantly downregulated MMP2, MMP9, ATG5, Beclin1, and LC3 II/I were detected in M-AMCs co-cultured with HAMSCs and HBMSCs than those co-cultured with HBMSCs (Fig. 7H–L). These findings showed that HAMSCs inhibited the role of HBMSCs in promoting the behaviors of co-cultured M-AMCs.

### HAMSCs promote cell behaviors of HBMSCs inhibited by M-AMCs

**Fig. 8:**
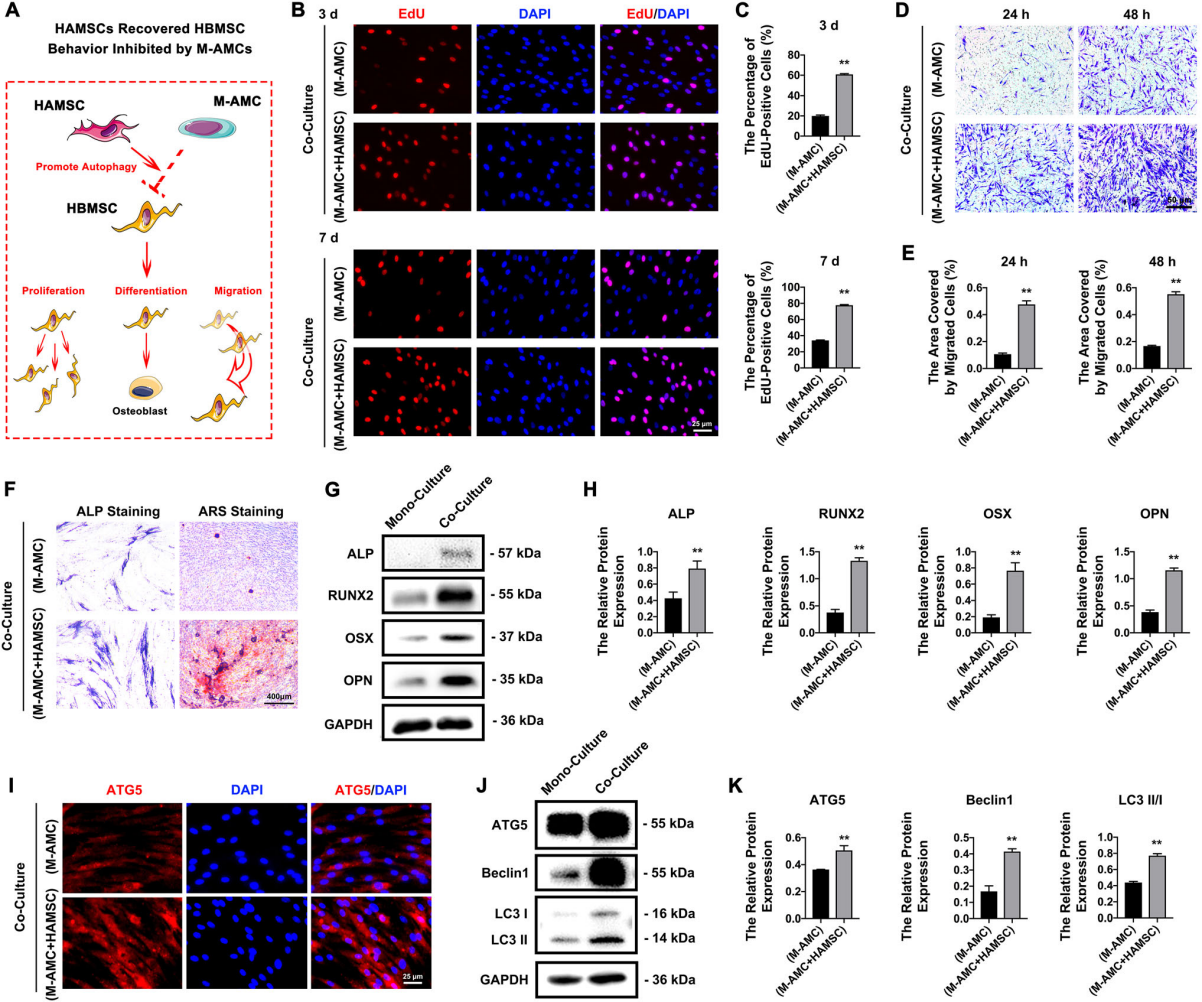
HAMSCs recover cell behaviors in HBMSCs inhibited by M-AMCs. **A** schematic diagram of HAMSCs in promoting the proliferation, invasion and migration of HBMSCs promoted by M-AMCs. **B**, **C** EdU-positive HBMSCs (red) in HBMSCs/M-AMCs co-culture system and HAMSCs/HBMSCs/M-AMCs co-culture system (scale bar: 25 μm). Cell nuclei were stained with DAPI (blue). **D**, **E** Transwell migration assay of HBMSCs in HBMSCs/M-AMCs co-culture system and HAMSCs/HBMSCs/M-AMCs co-culture system (scale bar: 50 μm). **F** ALP and ARS staining of HBMSCs in HBMSCs/M-AMCs co-culture system and HAMSCs/HBMSCs/M-AMCs co-culture system (scale bar: 50 μm). **G**, **H** Protein expressions of ALP, RUNX2, OSX and OPN in HBMSCs of HBMSCs/M-AMCs co-culture system and HAMSCs/HBMSCs/M-AMCs co-culture system. **I** Immunofluorescent staining of ATG5 (red) in HBMSCs of HBMSCs/M-AMCs co-culture system and HAMSCs/HBMSCs/M-AMCs co-culture system (scale bar: 25 μm). Cell nuclei were stained with DAPI (blue). **J**, **K** Protein expressions of ATG5, LC3 II/I and Beclin1 in HBMSCs of HBMSCs/M-AMCs co-culture system and HAMSCs/HBMSCs/M-AMCs co-culture system. **p < 0.01

We detected a significantly higher percentage of EdU-positive HBMSCs (Fig. 8B, C) and higher migratory (Fig. 8D, E) and more pronounced ALP and ARS staining (Fig. 8F) of co-cultured HBMSCs with HAMSCs and M-AMCs, compared with those of HBMSCs co-cultured with M-AMCs. ALP, RUNX2, OSX, OPN, ATG5, Beclin1, and LC3 II/I were significantly upregulated in HBMSCs co-cultured with HAMSCs and M-AMCs, compared to those in HBMSCs co-cultured with M-AMCs (Fig. 8G–K). Collectively, HAMSCs enhanced the role of M-AMCs in inhibiting the behaviors of co-cultured HBMSCs.

### HAMSCs promote bone regeneration *in vivo*

**Fig. 9:**
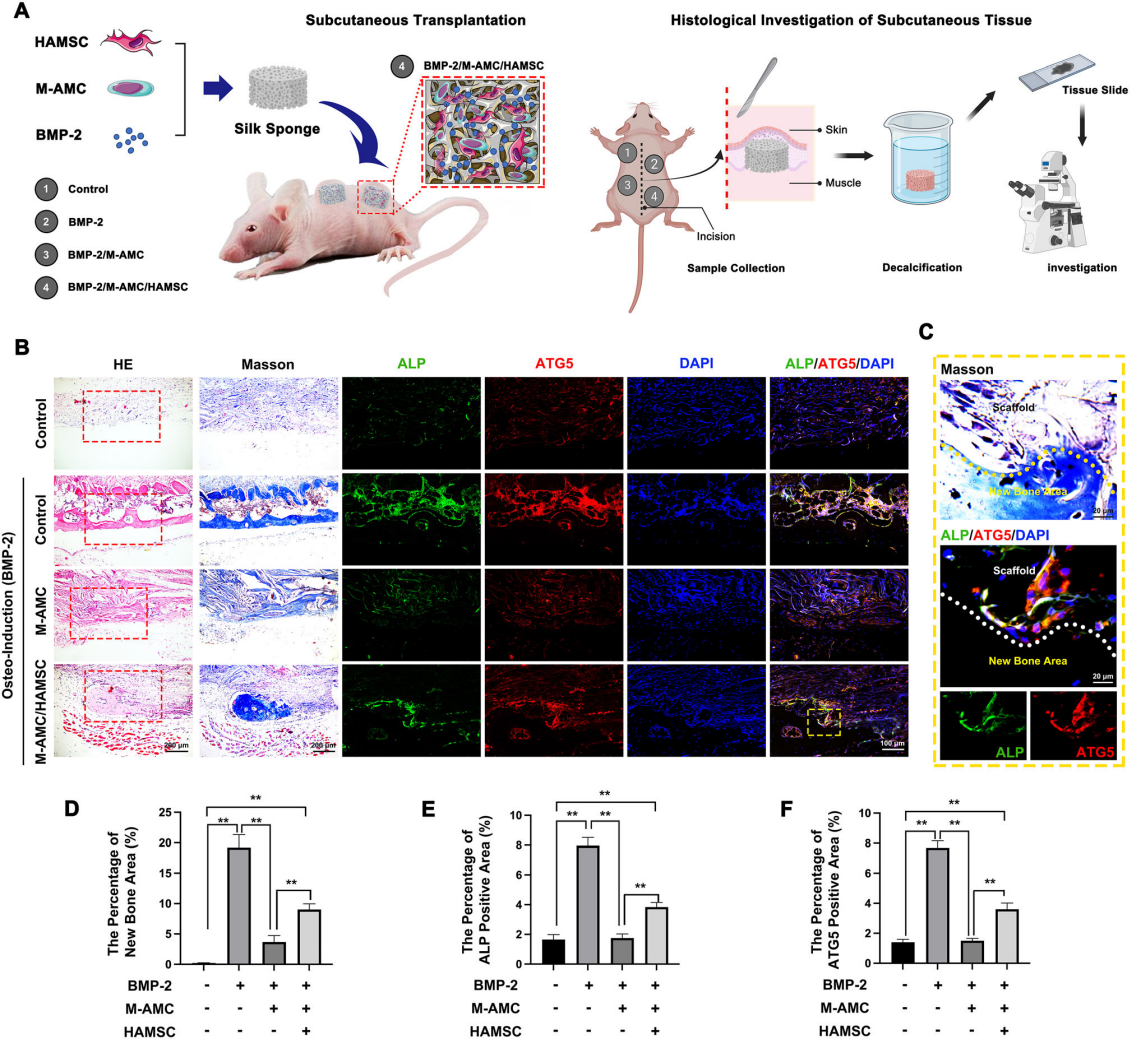
HAMSCs promote bone regeneration *in vivo*. **A** A schematic diagram of nude mice subcutaneously transplanted with silk scaffolds containing blank control, BMP-2, M-AMCs and M-AMCs co-cultured with HAMSCs (n = 6 per group). **B** H&E staining, Masson’s trichrome staining and immunofluorescence of ALP (green), ATG5 (red) and nuclei (blue) in harvested tissue blocks of silk scaffolds (scale bar: 200 μm in H&E and Masson’s trichrome staining; 100 μm in immunofluorescent staining). **C** Co-localization of ALP and ATG5 in the new bone area (scale bar: 100 μm). **D**–**F** Percentage of new bone area **D** ALP-positive new bone area (**E**) and ATG5-positive new bone area (**F**). **p < 0.01

To further explore the *in vivo* role of HAMSCs in stimulating bone regeneration by suppressing cell behaviors of M-AMCs, we assessed macrophage infiltration in nude mice of the four groups at 4 weeks after scaffold transplantation (Fig. 9A). New bone formation was significantly inhibited in mice transplanted with silk scaffolds containing M-AMCs. The area of new bones was larger in mice transplanted with silk scaffolds containing both HAMSCs and M-AMCs than in other groups, suggesting that HAMSCs promoted osteogenesis in bone defects (Fig. 9B, D). Interestingly, ALP was co-localized with ATG5 in the new bone area (Fig. 9C). The percentages of ATG5-positive area and ALP-positive area were significantly higher in mice transplanted with silk scaffolds containing both HAMSCs and M-AMCs than those with silk scaffolds containing M-AMCs, indicating the involvement of enhanced autophagy in HAMSCs-induced bone regeneration (Fig. 9E, F).

## Discussion

Tumor cells transform the TME to orchestrate invasion, metastasis, and therapeutic resistance [16]. In ameloblastoma, this orchestration remains poorly delineated, particularly regarding the role of autophagy, a conserved catabolic process that preserves cellular homeostasis under metabolic or oxidative stresses [17]. Our study filled this knowledge gap by identifying a novel mechanism through which HAMSCs disrupt the crosstalk of autophagy with the TME in the ameloblastoma. Specifically, HAMSCs selectively inhibit the autophagy in M-AMCs to suppress tumorigenesis, while restoring the osteogenic differentiation capacity of HBMSCs. This dual, context-dependent role provides new insights into both ameloblastoma biology and therapeutic development.

We first confirmed that basal autophagy was elevated in the stroma of ameloblastoma, aligning with emerging evidence that autophagy supports the stem-like properties and apoptotic resistance of M-AMCs [18]. Our co-culture experiments revealed that M-AMCs impaired HBMSC proliferation, osteogenic differentiation, and migration by suppressing autophagy—an effect reminiscent of observations in multiple myeloma, where tumor cells hijack stromal autophagy to drive bone destruction [19, 20]. However, HAMSCs reversed this effect. In tri-culture systems (HAMSCs + M-AMCs + HBMSCs), they restored autophagic flux in HBMSCs (via upregulating ATG5 and Beclin1), while specifically inhibiting autophagy in M-AMCs. In this way, HAMSCs can promote stromal autophagy to support bone regeneration and dampen tumor cell autophagy to suppress tumor growth in ameloblastoma. This bidirectional regulation has not been described before, highlighting the innovation of our present study.

Mechanistically, this selectivity likely arises from HAMSCs’ paracrine signalling. Studies in hepatocellular carcinoma and glioma have shown that HAMSCs secrete bioactive factors, including exosomes and TGF-β, to modulate autophagy-related pathways, such as TGF/Smad and Beclin1/Bcel-2 [21–23]. Our data extended this paradigm to ameloblastoma. In M-AMCs, HAMSC-derived TGF-β1 activated the TGF/Smad signalling pathway in M-AMCs, which directly exerts an adverse regulatory effect on their basal autophagy [24, 25]; in HBMSCs, they upregulated Beclin1, suggesting a pro-autophagic effect to restore stromal function [26]. This context-dependent modulation distinguishes HAMSCs from non-selective autophagy inhibitors, like chloroquine, which often disrupt normal tissue homeostasis [27, 28], suggesting a profound therapeutic potential of HAMSCs in future clinical settings.

Notably, our findings also shed light on the dual role of HBMSCs in the ameloblastoma TME. HBMSCs promoted M-AMCs proliferation, migration, invasion, and autophagy, aligning with the tumor-promoting roles of stromal cells in lung adenocarcinoma but contrasting with their anti-tumor roles in triple-negative breast cancer. This duality underscores the complexity of the TME and the need for targeted interventions. Previous studies have shown that HAMSCs accelerate the regeneration of endogenous vascularized bones by enhancing the osteogenic differentiation of HBMSCs in bone defects [29]. During the osteogenic differentiation of BMSCs, Beclin1 and LCII/I are upregulated by HAMSCs, suggesting the involvement of cell autophagy [15]. In glioma tumors, intravenous administrations of HAMSCs decrease the tumor size and suppress tumor cell migration by impairing the Beclin1/ Bcl2 complex [30]. In the present study, however, HAMSCs acted as “regulators” of stromal-tumor crosstalk: abrogating HBMSCs’ pro-tumor effects on M-AMCs while reversing M-AMCs’ inhibitory effects on HBMSCs. This ability of HAMSCs to “reset” the TME balance has never been reported in previous stem cell-based therapies for odontogenic tumors.

*In vivo* models of ameloblastoma have been rarely constructed. Due to their slow growth, subcutaneous injections of M-AMCs can barely form the tumor xenografts[31]. Silk scaffolds were adopted as effective carriers of M-AMCs and BMP-2 in our study. BMP-2 is a potent inducer of osteogenesis, even in ectopic sites [32]. The induction of BMP-2 at a very low concentration of 0.30 mg/ml can achieve new bone formation in subcutaneous tissues [33]. Our data revealed that new bone formation induced by BMP-2 was significantly inhibited in mice transplanted with silk scaffolds containing M-AMCs, and this effect was then partially reversed by co-cultured HAMSCs. *In vivo* evidence consistently supported the role of HAMSCs in promoting bone regeneration, even under the inhibition of M-AMCs. We have proven the high possibility of HAMSCs-based therapies inhibiting the progression and recurrence of ameloblastoma while simultaneously promoting bone regeneration.

Limitations of our study include the use of subcutaneous xenografts, which do not fully replicate the microenvironment of the jawbone, which is prone to ameloblastoma invasiveness [34]. Future work should validate these findings in orthotopic models with extended observation periods, given the slow growth of ameloblastoma [35, 36]. Additionally, specific paracrine factors mediating HAMSCs’ effects should be identified to enhance the translational potential of our findings, as seen in other cancers where such factors have been harnessed for targeted therapy [12, 37].

In summary, our study is the first to identify that HAMSCs can selectively inhibit tumor cell autophagy and restore the osteogenic ability of HBMSCs in ameloblastoma. This dual role posits HAMSCs as a promising therapeutic candidate to suppress myoblastoma recurrence and enhance bone regeneration.

## Data Availability

Data supporting the findings of this study are available from the corresponding author upon reasonable request.
